# Non-contrast computed tomography of type A acute aortic dissection in patients with out-of-hospital cardiopulmonary arrest: a case series

**DOI:** 10.1093/ehjcr/ytz218

**Published:** 2019-12-06

**Authors:** Shinsuke Takeuchi, Yoshihiro Yamaguchi, Hideaki Yoshino

**Affiliations:** 1 Division of Cardiology, Second Department of Internal Medicine, Kyorin University School of Medicine, 6-20-2 Shinkawa, Mitaka-shi, Tokyo 181-8611, Japan; 2 Department of Trauma and Critical Care Medicine, Kyorin University School of Medicine, 6-20-2 Shinkawa, Mitaka-shi, Tokyo 181-8611, Japan

**Keywords:** Aortic dissection, Out-of-hospital, Cardiopulmonary arrest, Computed tomography, Case series

## Abstract

**Background:**

The prognosis of patients admitted for acute aortic dissection (AAD) has remarkably improved. However, we must also consider out-of-hospital cardiopulmonary arrest (OHCPA) patients while assessing the prognosis. In recent years, autopsy imaging has become more common as an alternative to conventional autopsy. Therefore, we reviewed our OHCPA patients with type A AAD using acute phase non-contrast computed tomography (CT).

**Case summary:**

Here, we report a case series of three patients who developed OHCPA and were diagnosed with type A AAD using non-contrast CT. Although the direct causes of death varied in each case, we could easily determine the direct causes of death from clinical course of the condition and from non-contrast CT.

**Discussion:**

Although non-contrast CT does not completely replace autopsy, if its convenience and non-invasiveness make it possible for more patients to undergo the procedure, the real prognosis (including morbidity and mortality) may be better understood. Therefore, we considered it significant to use non-contrast CT for investigating the cause of sudden death.

**Learning points**

- There are many cases of acute aortic dissection with out-of-hospital cardiopulmonary arrest that can be diagnosed using non-contrast computed tomography (CT), and it is often possible to estimate the direct cause of death.
- Acute phase CT cannot completely replace conventional autopsy.
- However, it is useful because it can be performed in many cases because of its convenience and non-invasiveness.

## Introduction

With the progress in medical technology, the prognosis of patients admitted for acute aortic dissection (AAD) has remarkably improved;^1^ however, it may demonstrate a high mortality rate, because many cases with AAD are considered as patients with out-of-hospital cardiopulmonary arrest (OHCPA).^2^ Thus, the exact causes of death might often be unclear. The incidence of AAD, as known currently, has been mainly calculated from hospitalized patients. Therefore, it is extremely important to investigate AAD cases, including OHCPA patients to accurately assess the prognosis of AAD cases.

The aim of our report was to validate the findings of non-contrast computed tomography (CT) in three cases of AAD with OHCPA and to propose that CT diagnosis be actively performed as a way of determining the cause of death in OHCPA patients. The CT images were acquired on an 80-slice multidetector scanner (Aquilion ONE, Canon Medical Systems, Tochigi, Japan) with a thickness of 5 mm, tube voltage of 120 kVp, and auto exposure control. The CT values for bloody pericardial effusion and haematoma of the thoracic cavity are shown in Hounsfield units (HUs).

## Timeline

**Table:** 

Patient 1	
16 min prior	Disturbance of consciousness, abnormal respiration
0 min [cardiopulmonary arrest CPA)]	Bystander cardiopulmonary resuscitation (CPR) performed by his family
3 min later	Emergency medical service (EMS) arrival at the scene, initial electrocardiogram (ECG): pulseless electrical activity (PEA)
37 min later	Arrival at the hospital
58 min later	Discontinuation of CPR
77 min later	Computed tomography (CT) performed
Patient 2	
[Day of admission]	
About 15 min prior	Discomfort
Unknown (CPA)	Witness (+), Bystander CPR (−)
0 min (EMS arrival at the scene)	CPR initiated by EMS, Initial ECG: PEA
38 min later	Return of spontaneous circulation
[Hospital Day 2]	(Emergency operation not performed)
0 min (CPA)	CPR started, Initial ECG: PEA
28 min later	Discontinuation of CPR
44 min later	CT performed
Patient 3	
0 min (CPA)	Collapsed suddenly in front of her family (Bystander CPR +)
9 min later	EMS arrival at the scene, Initial ECG: ventricular fibrillation (VF)
40 min later	Arrival at hospital
69 min later	Venoarterial extracorporeal membrane oxygenation (VA-ECMO) established because of incessant VF
195 min later	CT performed, (impossible systematic circulation maintenance)

## Case presentation

### Patient 1

Our first patient, a man in his early 80s, presented with hypertension, sequelae of left hemiparesis owing to cerebral haemorrhage, and severe chronic obstructive pulmonary disease. Home oxygen therapy was introduced, and he was provided with home medical care.

One evening, his family noticed deterioration of his consciousness and stertorous respiration while at home. Just prior to arrival of the emergency medical services (EMS), cardiopulmonary arrest (CPA) occurred, and bystander cardiopulmonary resuscitation (CPR) was initiated by the family.

The initial electrocardiogram (ECG) showed pulseless electrical activity (PEA) at the time of arrival of the EMS. Thereafter, CPR was continued for 55 min; however, the return of spontaneous circulation (ROSC) could not be achieved.

**Figure 1:**
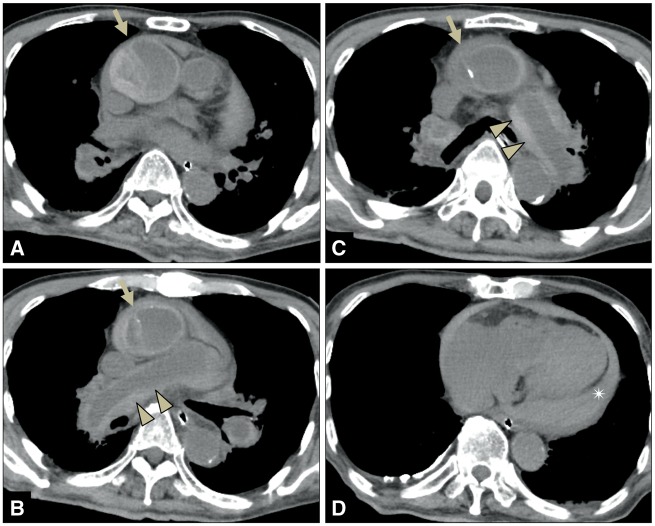
Computed tomography image of Patient 1 after the discontinuation of cardiopulmonary resuscitation shows an intimomedial flap localized to the ascending aorta (*A*-arrow), inward shift of the calcified intima (*B*/*C*-arrows), increased bilateral pulmonary artery wall attenuation (*B*/*C*-arrowheads), and large amount of bloody pericardial effusion (*D*-asterisk). The computed tomography values of bloody pericardial effusion were 40–52 HUs.

Computed tomography imaging after discontinuation of CPR showed an intimomedial flap localized to the ascending aorta and an inward shift of the calcified intima into a portion of the aortic lesion (*Figure 1*). The diameter of the false lumen was 26.6 mm from the maximum short axis diameter of 48.0 mm, and the enlarged false lumen was pushed against the true lumen. Increased attenuation was seen along the wall of the bilateral pulmonary artery, probably owing to ‘haemorrhagic infiltration through the common aortopulmonary adventitia’^3^ when the aortic rupture occurred. Moreover, a large amount of bloody pericardial effusion was confirmed, and the direct cause of death was considered to be cardiac tamponade.

### Patient 2

**Figure 2:**
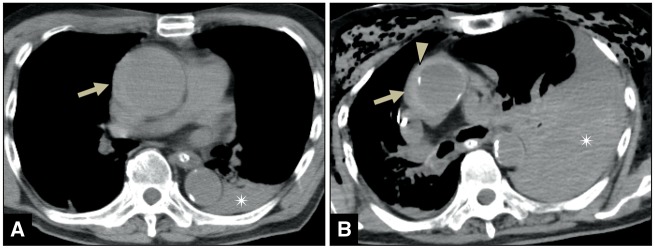
Computed tomography image of Patient 2 after return of spontaneous circulation (ROSC) on the day of admission shows crescentic hyperattenuating intramural fluid collection from the ascending aorta to the arch (*A*-arrow) and left pleural haematoma (*A*-asterisk). Computed tomography image after discontinuation of cardiopulmonary resuscitation on Day 2 of admission shows inward shift of the calcified intima (*B*-arrowhead), significant increase in the left pleural haematoma (*B*-asterisk), and crescentic intramural fluid collection revealing more hyperattenuation (*B*-arrow). The computed tomography values of left pleural haematoma were 33–43 HUs on admission and 45–60 HUs on Day 2 of admission.

A male patient in his early 80s presented with hypertension. He complained of discomfort while driving a car with his wife. The car slowly bumped onto the guardrail and stopped. Immediately, his wife requested EMS, and CPA was confirmed upon arrival of the EMS at the scene. The initial ECG at the scene showed PEA. After 38 min of CPR, ROSC was confirmed. Non-contrast CT imaging showed ‘crescentic hyperattenuating intramural fluid collection’^4^ from the ascending aorta to the arch, along with an inward shift of the calcified intima into a portion of the aortic lesion (*Figure 2*). In addition, bleeding into the left thoracic cavity was noted. An emergency operation was not performed owing to the poor prognosis regarding brain function. On Day 2 of admission, he developed CPA again, and CPR was performed immediately. However, he died without ROSC. The initial ECG obtained at the time of the CPA showed PEA, while that obtained after discontinuation of CPR showed a significant increase in the left pleural haematoma.

### Patient 3

**Figure 3:**
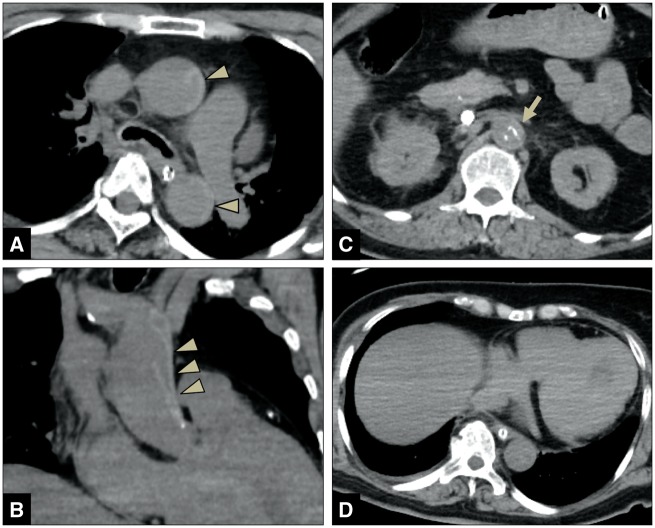
Computed tomography imaging of Patient 3 shows aortic dissection extending from the root of the ascending aorta to the common iliac artery. The true lumen from the ascending aorta to the descending thoracic aorta completely collapsed due to the enlarged false lumen (*A*/*B*-arrowheads) and inward shift of the calcified intima at the abdominal aorta (*C*-arrow). No findings, such as bloody pericardial effusion and haemothorax, suggesting a rupture of the aorta (*D*).

A female patient in her late 60s had a medical history of hypertension and diabetes. She collapsed in front of her family, and bystander CPR was performed. The initial ECG on arrival of the EMS at the scene showed ventricular fibrillation (VF). CPR was continued, and defibrillation was repeatedly performed. Since VF was sustained even after arrival at the emergency room, venoarterial extracorporeal membrane oxygenation was established. However, it was impossible to maintain the systemic circulation. The CT showed aortic dissection extending from the root of the ascending aorta to the common iliac artery (*Figure 3*). Although there were no findings suggestive of a rupture of the aorta, the true lumen from the ascending aorta to the descending thoracic aorta was pushed and completely collapsed because of the enlarged false lumen. Based on these findings, myocardial ischaemia owing to occlusion of the coronary artery ostium was considered to be the direct cause of death.

## Discussion

The three patients with OHCPA we experienced could be diagnosed with type A AAD and the direct causes of death could be easily determined using the clinical course of the condition and non-contrast CT findings.

In past reports,^5–7^ the diagnostic criteria for AAD by non-contrast CT performed after CPA were as follows: (a) intimomedial flap; (b) inward shift of the calcified intima; (c) double sedimentation in the true and false lumen caused by hypostasis; and (d) the presence of intramural haematoma (IMH). In each paper, either criteria a, b, and c or criteria a, b, and d were adopted. Additionally, IMH is precisely defined as ‘a variant of dissection characterized by the absence of an intimal tear’ caused by haemorrhage of the aortic vasa vasorum into the aortic wall.^8^ However, as it is usually difficult to distinguish the tear itself using non-contrast CT, we interpreted the criterion (d) as ‘crescentic hyperattenuating intramural fluid collection’.^4^ The ‘intimomedial flap’ was detected in Cases 1 and 3, the ‘inward shift of the calcified intima’ was observed in all cases, and the ‘crescentic hyperattenuating intramural fluid collection’ was only detected in Case 2. On the other hand, ‘double sedimentation in the true and false lumen caused by hypostasis’ was not observed in any case. This is considered to be the reason for marginal influence of post-mortem changes in all cases because CT was performed immediately after discontinuation of CPR. In fact, few other post-mortem changes except for hypostasis were also observed in these patients.^9^^,^^10^ Furthermore, we diagnosed the direct causes of death in the three cases as follows: Case 1, cardiac tamponade because of aortic rupture into the pericardium; Case 2, left pleural haematoma due to aortic rupture into the left pleural cavity; and Case 3, myocardial ischaemia due to coronary artery occlusion as incessant VF and complete collapsed true lumen were observed without evidence of aortic rupture. All three cases described above could be easily diagnosed with AAD, and the associated direct causes of death could also be diagnosed.

Ampanozi *et al*.^5^ reported that out of 33 cases diagnosed with AAD at autopsy, 25 (about 76%) could be diagnosed with AAD using non-contrast CT performed before autopsy. In their other study, they also reported that among 11 cases of AAD with bloody pericardial effusion, 8 cases could be correctly diagnosed using non-contrast CT performed before autopsy. At the time, the sensitivity and specificity were 72.7% and 100%, respectively.^6^ Although the number of cases is small, these reports indicate that non-contrast CT alone is insufficient to diagnose AAD.

In fact, when using non-contrast CT, post-mortem changes such as hypostasis can lead to false positives. Moreover, severe collapse of the aorta due to aortic rupture can lead to a false negative. In such cases, it is necessary to perform other imaging procedures, such as CT angiography^11^ or MRI,^12^ and autopsy because diagnosis of AAD with non-contrast CT alone is particularly difficult.

In past reports based on autopsy,^13^^,^^14^ the incidence of AAD in patients with non-traumatic OHCPA was ∼3%. However, Tanaka *et al*. investigated the cause of death with acute phase CT. Thereafter, they reported that the type A AAD patients constituted ∼7% of the non-traumatic OHCPA cases. Moreover, Moriwaki *et al*.^15^ reported that AAD (including both type A and type B) cases were found in 9.17% of non-traumatic OHCPA cases, mainly in the study that conducted diagnoses by perimortem CT. The incidence of AAD in their report was much higher than that in the previous reports based on autopsy.

Although autopsy is the most reliable procedure for identifying the cause of sudden death, it is difficult to cover all cases owing to staff shortages, potential risks, and ethical and religious issues. Therefore, diagnostic imaging, in particular non-contrast CT, is considered to be superior to autopsy in terms of convenience and non-invasiveness. Although the sensitivity of non-contrast CT is insufficient, the technique is considered extremely useful to understand the morbidity and mortality associated with AAD.

Thus, this case series presented three cases of AAD that showed typical mechanisms leading to death. We could determine the presence of AAD and its serious complications using non-contrast CT alone. There are not enough reports on the effectiveness of perimortem imaging including non-contrast CT, and few studies have investigated the detailed cause of death based on the features of the images. Our study, which is validated based on the features of detailed images, is considered to be useful for the diagnosis of AAD cases with OHCPA and the epidemiological study of AAD using non-contrast CT.

## Lead author biography

**Figure:**
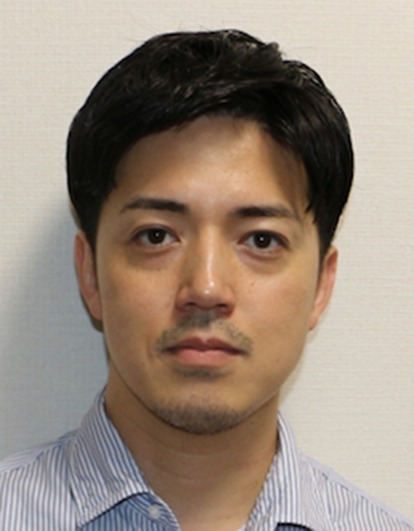


Shinsuke Takeuchi graduated from Kyorin University Medical School in 2009. He have been at a Medical Staff (Cardiologist) at the Division of Cardiology, Second Department of Internal Medicine, Kyorin University School of Medicine, since 2015. 

## Supplementary material

Supplementary material is available at *European Heart Journal - Case Reports* online.

**Slide sets:** A fully edited slide set detailing this case and suitable for local presentation is available online as Supplementary data.

**Consent:** Consent for publication was not obtained by the authors for the patients in this case series. However, the authors provided a means of opting out to the family of the deceased. Every effort has been made to ensure that patients are not identifiable in this publication.

**Conflict of interest:** none declared.

## Supplementary Material

ytz218_Supplementary_Slide_Set
