# Supplementary material for: Non-contrast computed tomography of type A acute aortic dissection in patients with out-of-hospital cardiopulmonary arrest: a case series
Source: Eur Heart J Case Rep. 2019 Dec 6;3(4):1–5. doi: 10.1093/ehjcr/ytz218 (PMC6939809; doi:10.1093/ehjcr/ytz218)
Supplement: ytz218_Supplementary_Slide_Set [file ytz218_supplementary_slide_set.pptx]

## Slide 1
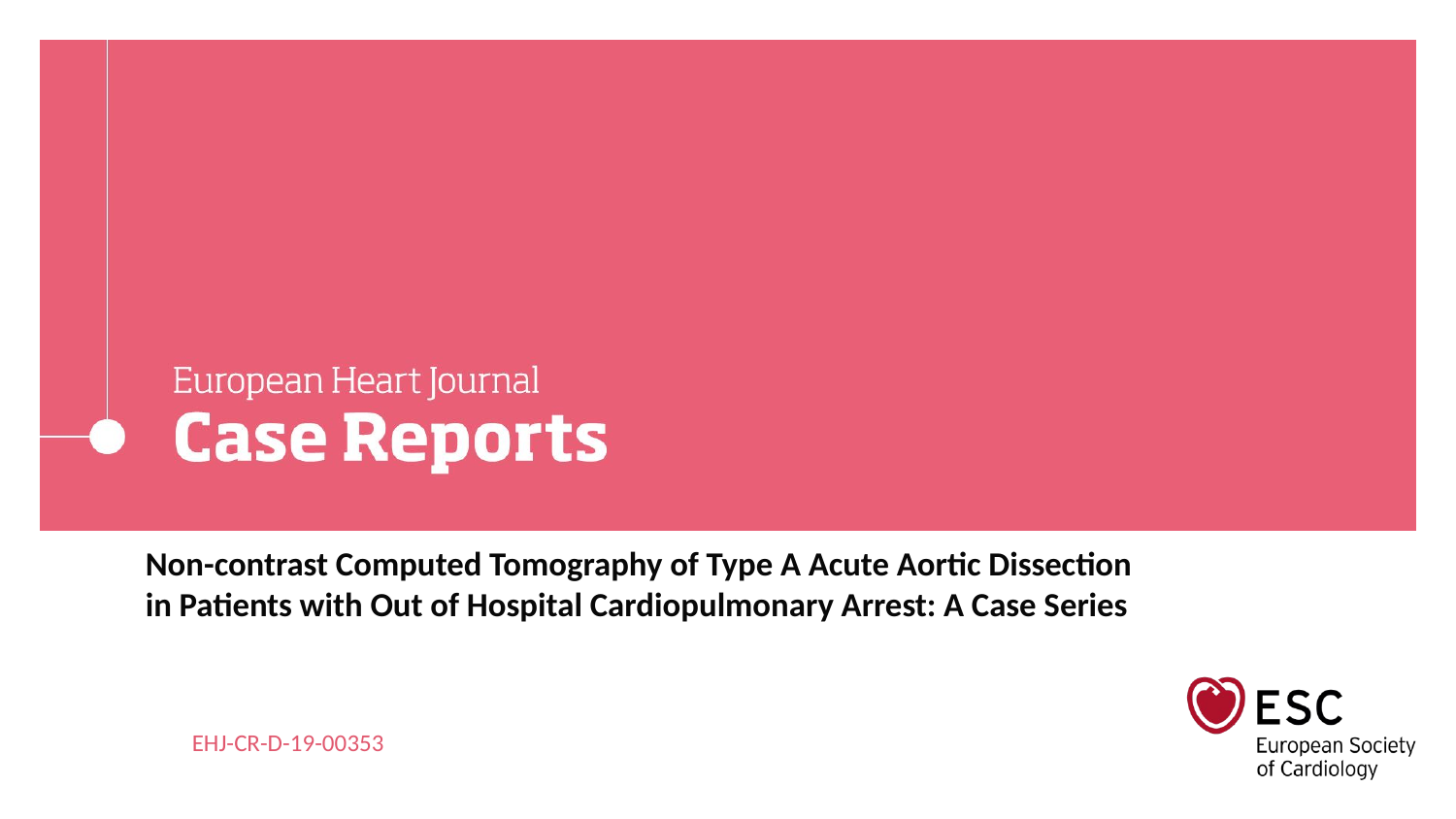

# Non-contrast Computed Tomography of Type A Acute Aortic Dissection in Patients with Out of Hospital Cardiopulmonary Arrest: A Case Series
EHJ-CR-D-19-00353

## Slide 2
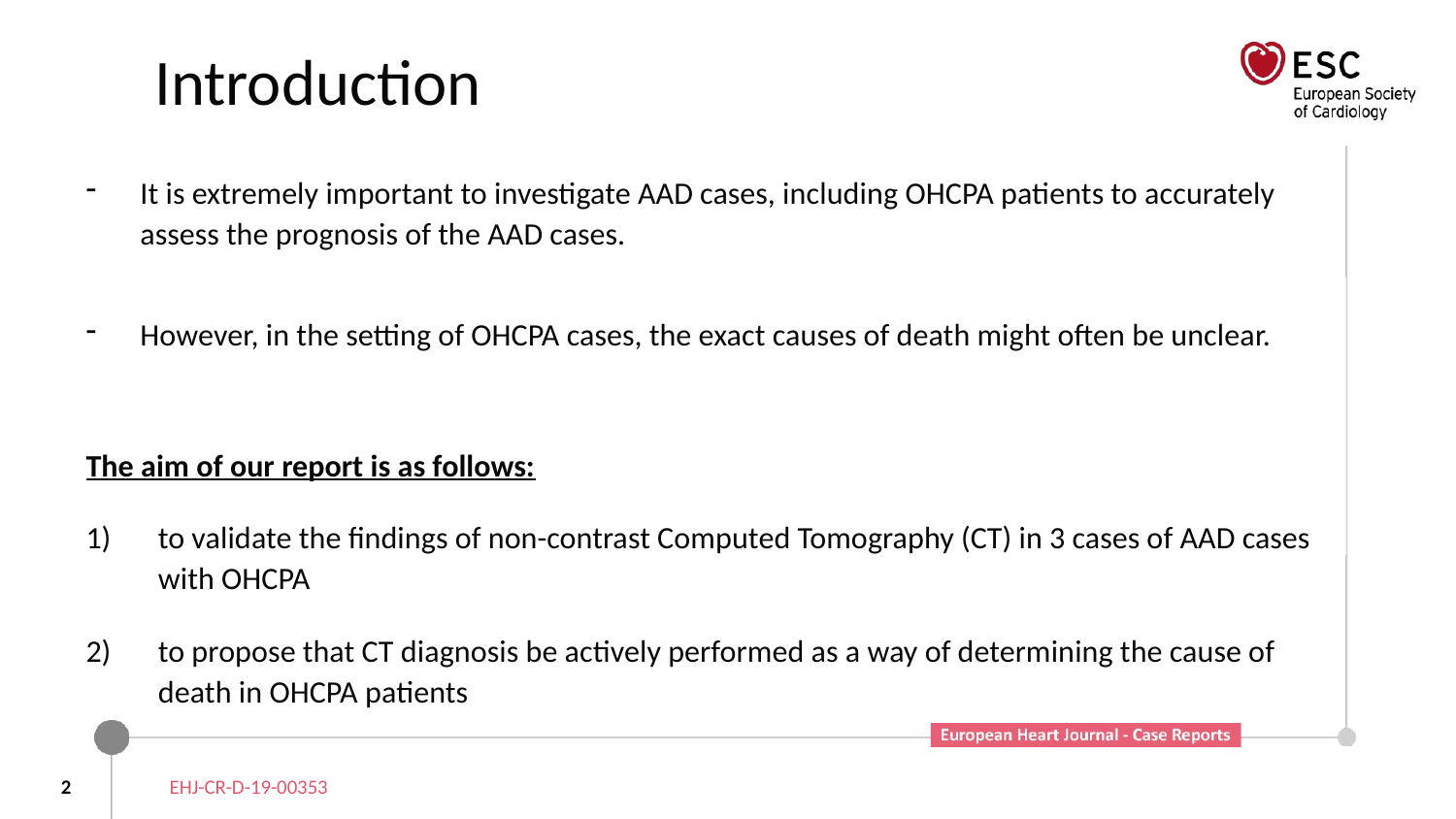

# Introduction
It is extremely important to investigate AAD cases, including OHCPA patients to accurately assess the prognosis of the AAD cases.
However, in the setting of OHCPA cases, the exact causes of death might often be unclear.
The aim of our report is as follows:
to validate the findings of non-contrast Computed Tomography (CT) in 3 cases of AAD cases with OHCPA
to propose that CT diagnosis be actively performed as a way of determining the cause of death in OHCPA patients
2
EHJ-CR-D-19-00353

## Slide 3
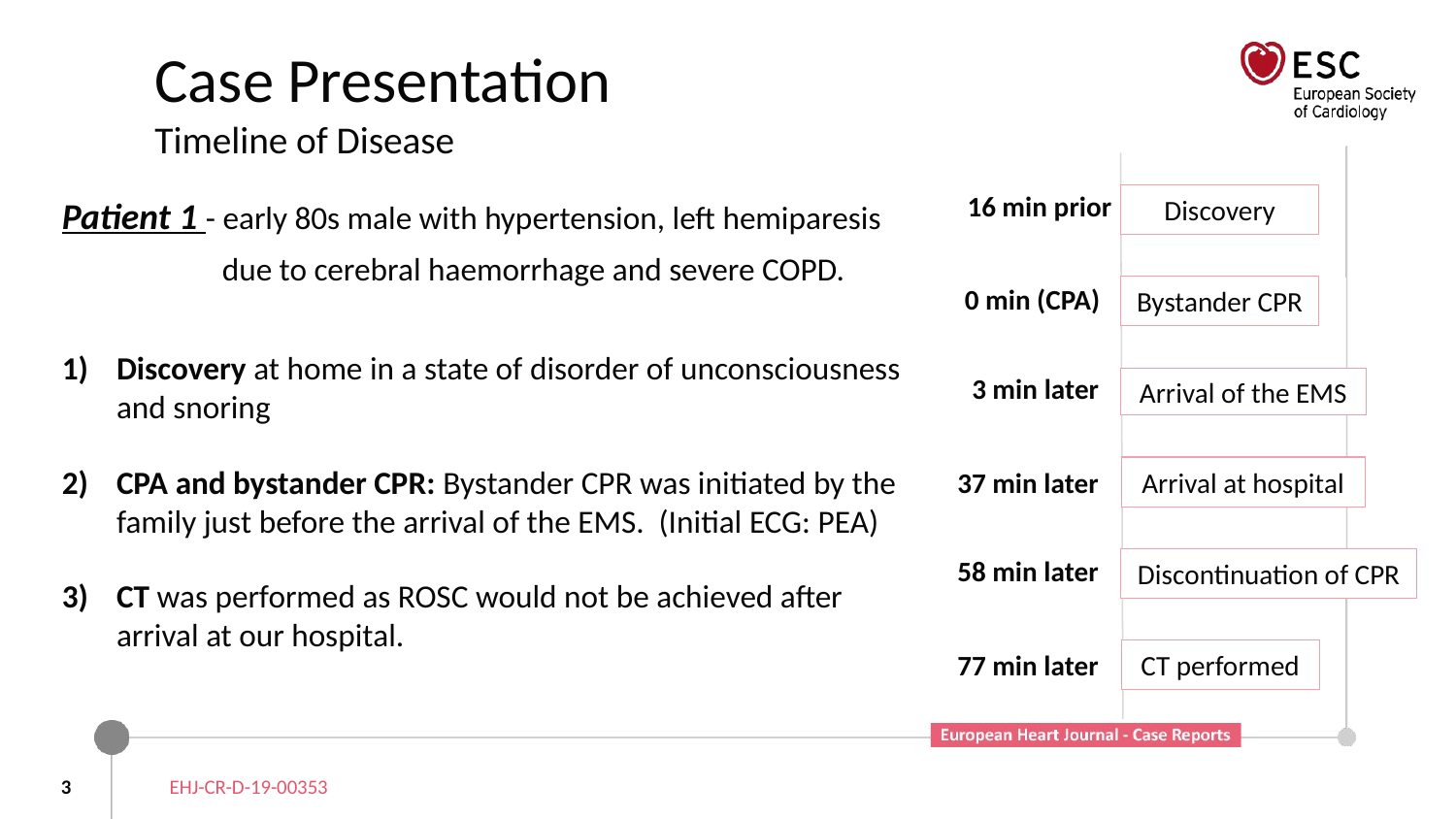

# Case Presentation Timeline of Disease
 16 min prior
Discovery
Patient 1 - early 80s male with hypertension, left hemiparesis
 due to cerebral haemorrhage and severe COPD.
Discovery at home in a state of disorder of unconsciousness and snoring
CPA and bystander CPR: Bystander CPR was initiated by the family just before the arrival of the EMS. (Initial ECG: PEA)
CT was performed as ROSC would not be achieved after arrival at our hospital.
0 min (CPA)
Bystander CPR
3 min later
Arrival of the EMS
Arrival at hospital
37 min later
58 min later
Discontinuation of CPR
CT performed
77 min later
3
EHJ-CR-D-19-00353

## Slide 4
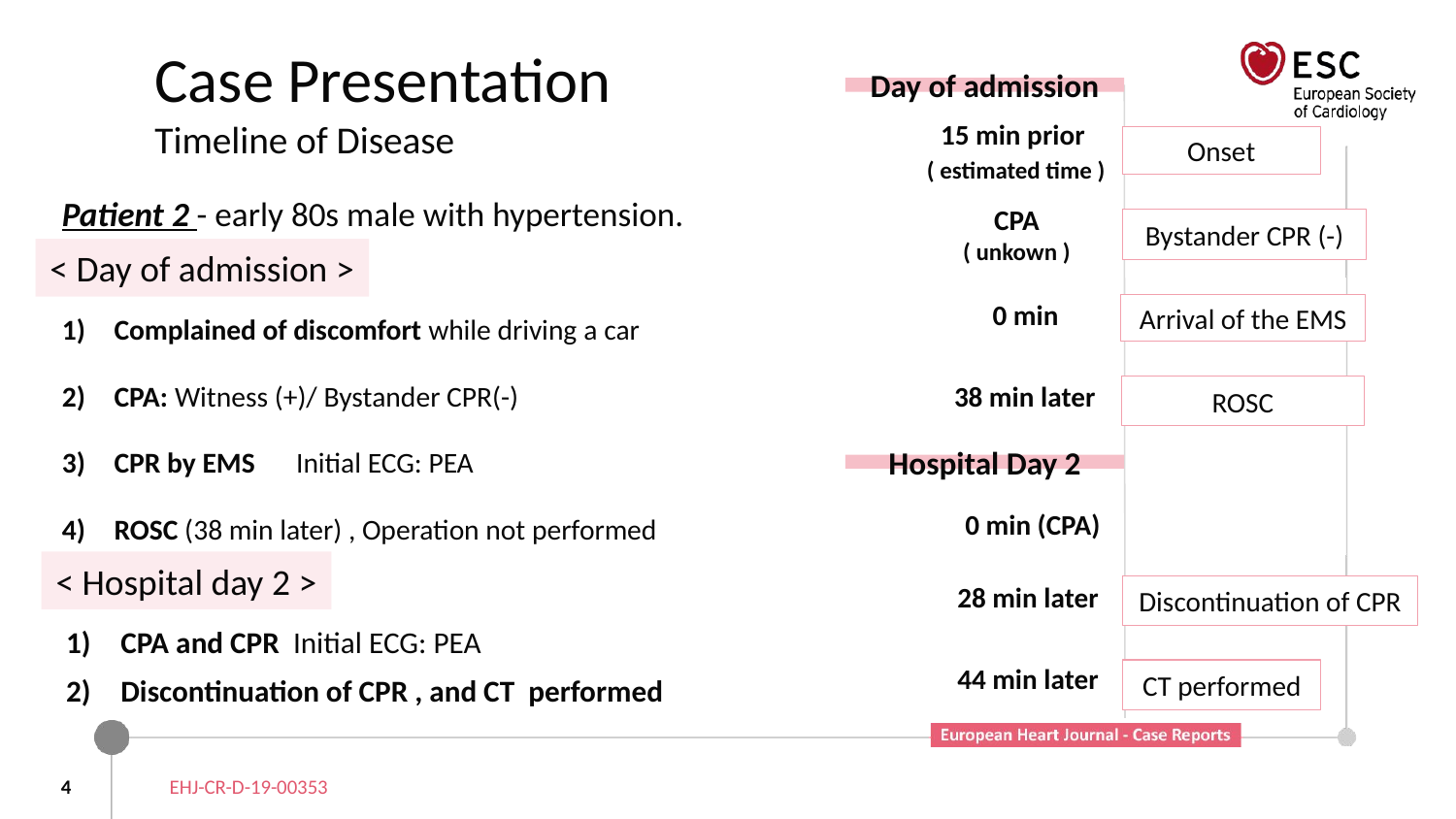

# Case Presentation Timeline of Disease
Day of admission
15 min prior
 ( estimated time )
Onset
Patient 2 - early 80s male with hypertension.
Complained of discomfort while driving a car
CPA: Witness (+)/ Bystander CPR(-)
CPR by EMS　Initial ECG: PEA
ROSC (38 min later) , Operation not performed
CPA
( unkown )
Bystander CPR (-)
< Day of admission >
0 min
Arrival of the EMS
38 min later
ROSC
Hospital Day 2
0 min (CPA)
< Hospital day 2 >
28 min later
Discontinuation of CPR
CPA and CPR Initial ECG: PEA
Discontinuation of CPR , and CT performed
44 min later
CT performed
4
EHJ-CR-D-19-00353

## Slide 5
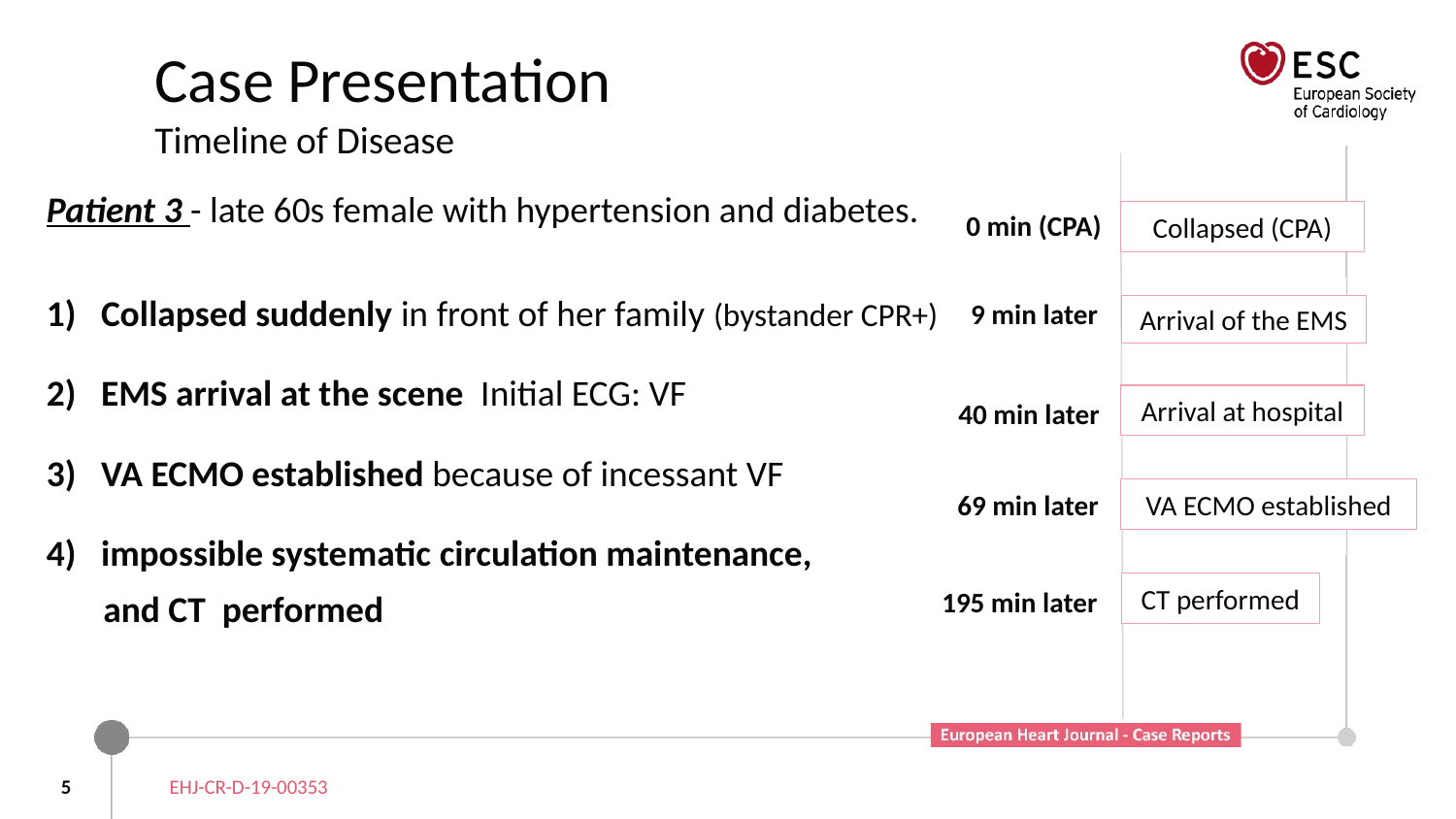

# Case Presentation Timeline of Disease
Patient 3 - late 60s female with hypertension and diabetes.
Collapsed suddenly in front of her family (bystander CPR+)
EMS arrival at the scene Initial ECG: VF
VA ECMO established because of incessant VF
impossible systematic circulation maintenance,
and CT performed
0 min (CPA)
Collapsed (CPA)
9 min later
Arrival of the EMS
Arrival at hospital
 40 min later
VA ECMO established
69 min later
CT performed
195 min later
5
EHJ-CR-D-19-00353

## Slide 6
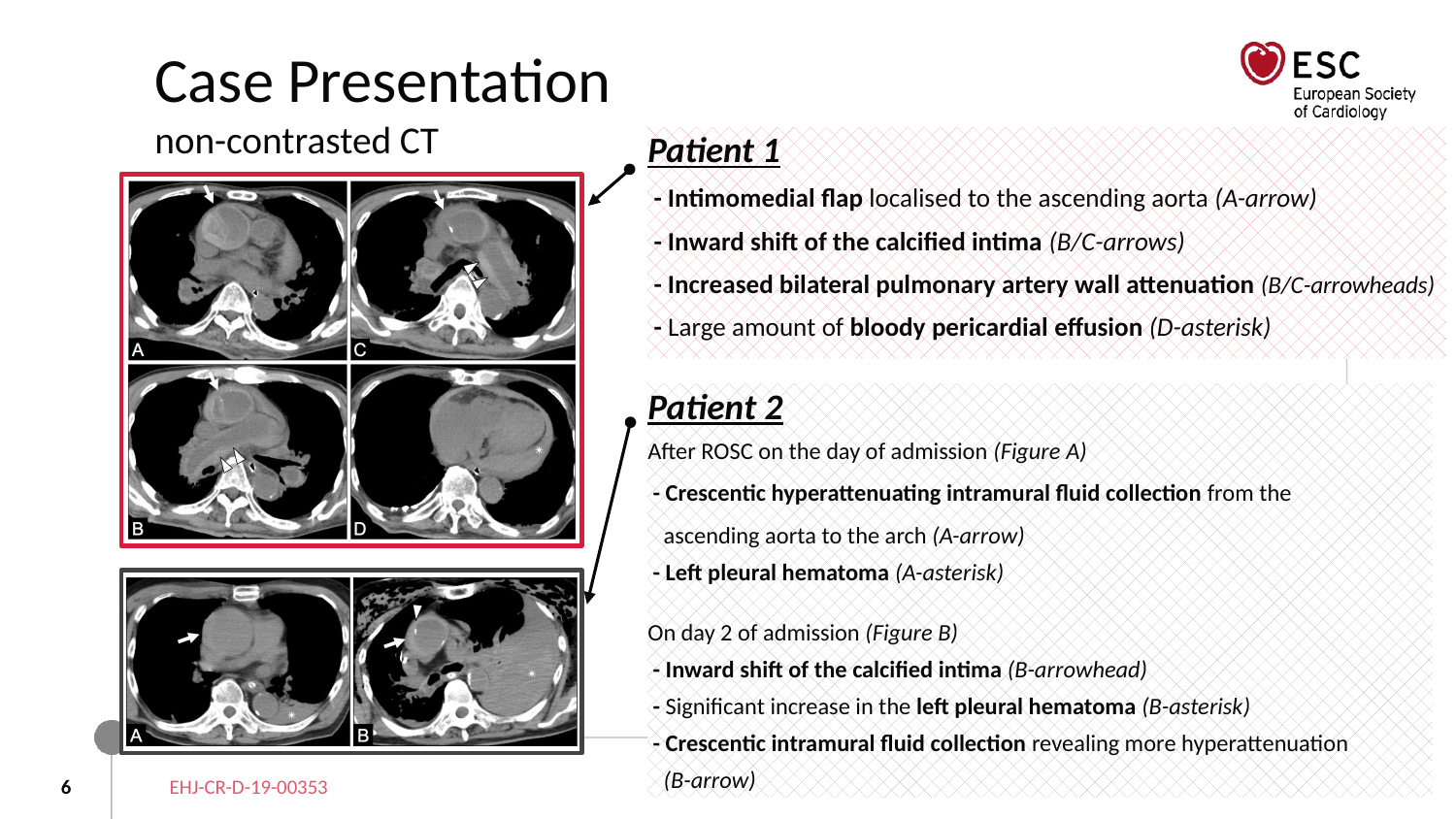

# Case Presentation non-contrasted CT
Patient 1
 - Intimomedial flap localised to the ascending aorta (A-arrow)
 - Inward shift of the calcified intima (B/C-arrows)
 - Increased bilateral pulmonary artery wall attenuation (B/C-arrowheads)
 - Large amount of bloody pericardial effusion (D-asterisk)
Patient 2
After ROSC on the day of admission (Figure A)
 - Crescentic hyperattenuating intramural fluid collection from the
 ascending aorta to the arch (A-arrow)
 - Left pleural hematoma (A-asterisk)
On day 2 of admission (Figure B)
 - Inward shift of the calcified intima (B-arrowhead)
 - Significant increase in the left pleural hematoma (B-asterisk)
 - Crescentic intramural fluid collection revealing more hyperattenuation
 (B-arrow)
6
EHJ-CR-D-19-00353

## Slide 7
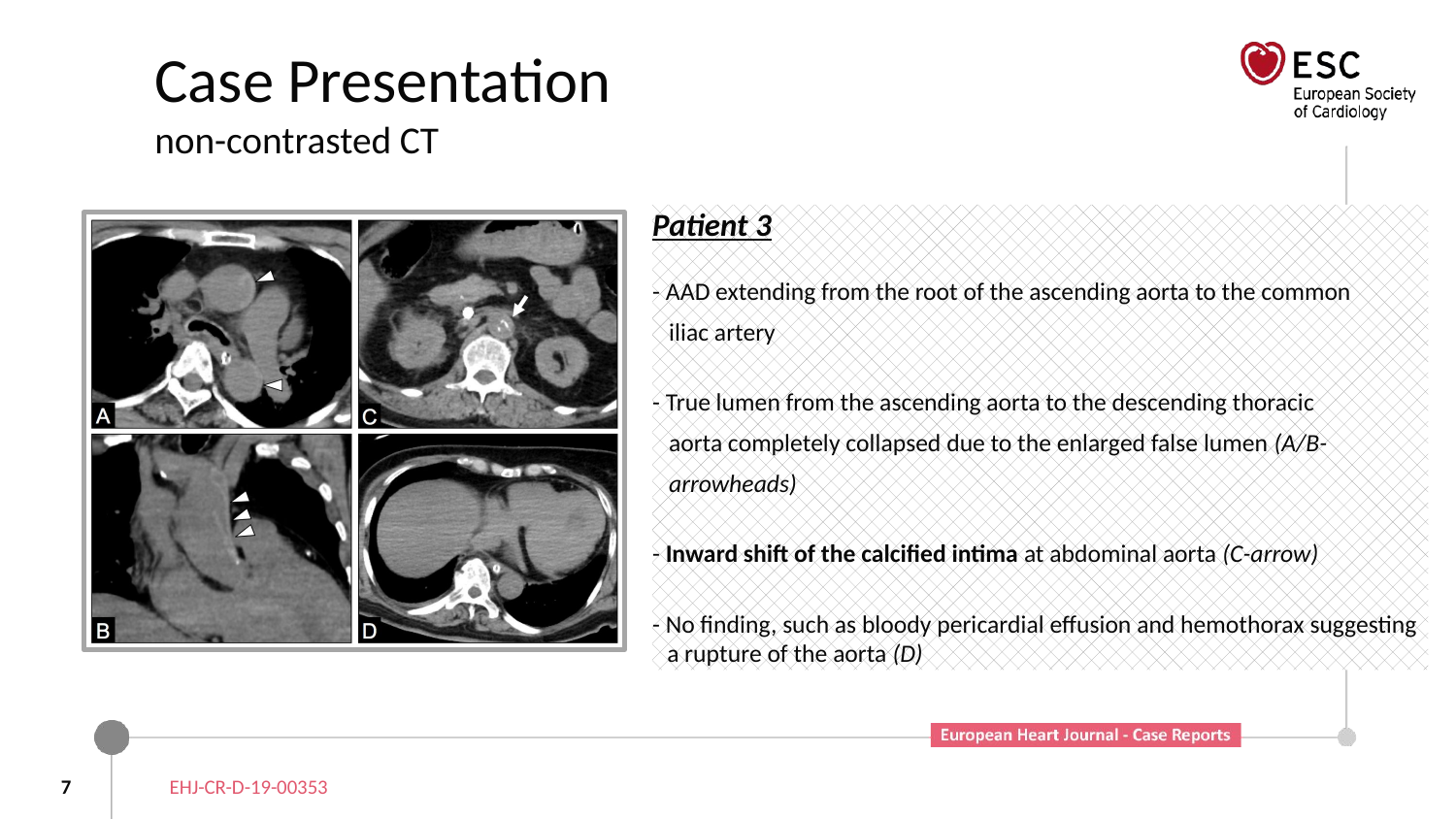

# Case Presentation non-contrasted CT
Patient 3
- AAD extending from the root of the ascending aorta to the common
 iliac artery
- True lumen from the ascending aorta to the descending thoracic
 aorta completely collapsed due to the enlarged false lumen (A/B-
 arrowheads)
- Inward shift of the calcified intima at abdominal aorta (C-arrow)
- No finding, such as bloody pericardial effusion and hemothorax suggesting a rupture of the aorta (D)
7
EHJ-CR-D-19-00353

## Slide 8
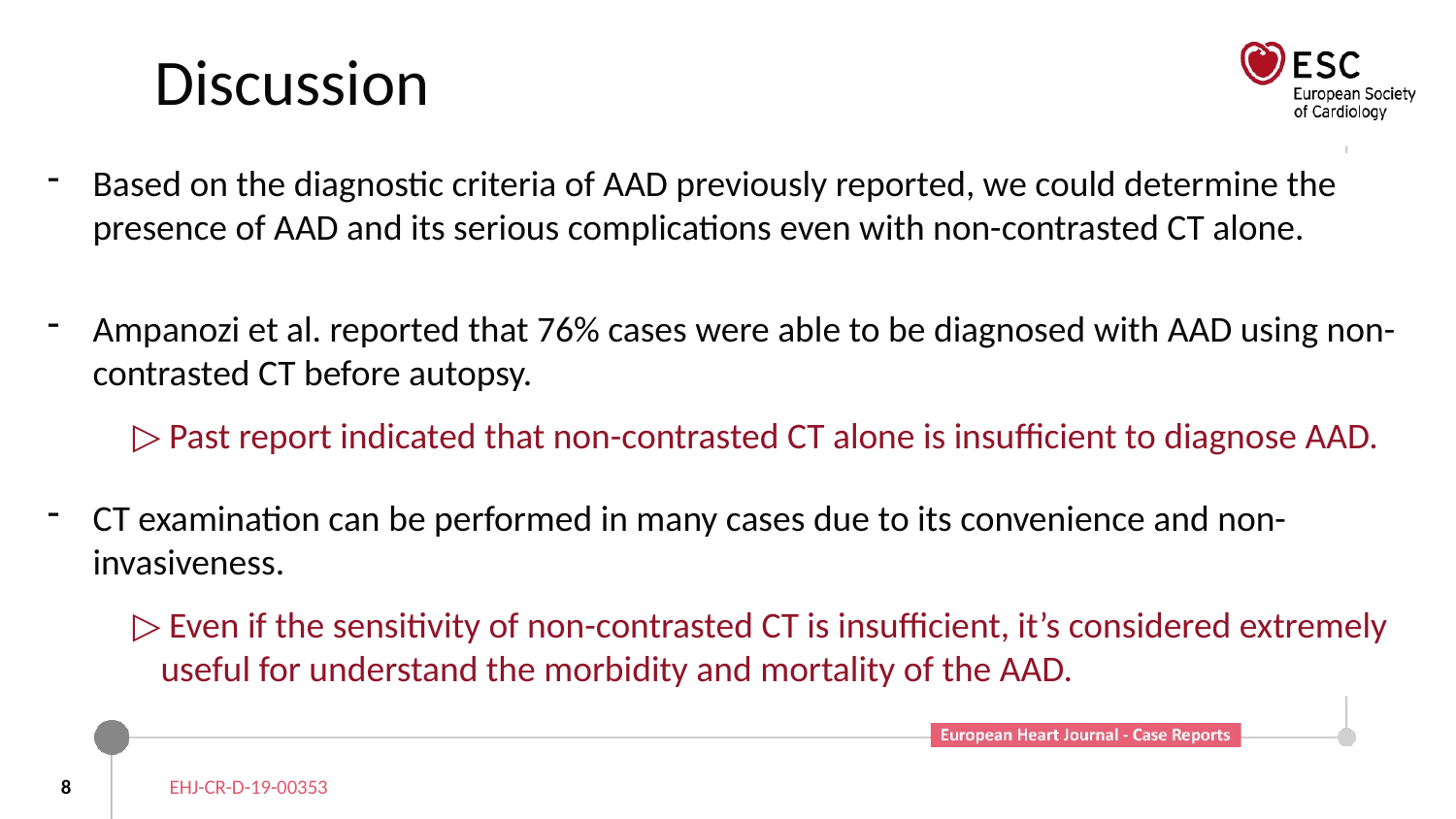

# Discussion
Based on the diagnostic criteria of AAD previously reported, we could determine the presence of AAD and its serious complications even with non-contrasted CT alone.
Ampanozi et al. reported that 76% cases were able to be diagnosed with AAD using non-contrasted CT before autopsy.
▷ Past report indicated that non-contrasted CT alone is insufficient to diagnose AAD.
CT examination can be performed in many cases due to its convenience and non-invasiveness.
▷ Even if the sensitivity of non-contrasted CT is insufficient, it’s considered extremely useful for understand the morbidity and mortality of the AAD.
8
EHJ-CR-D-19-00353

## Slide 9
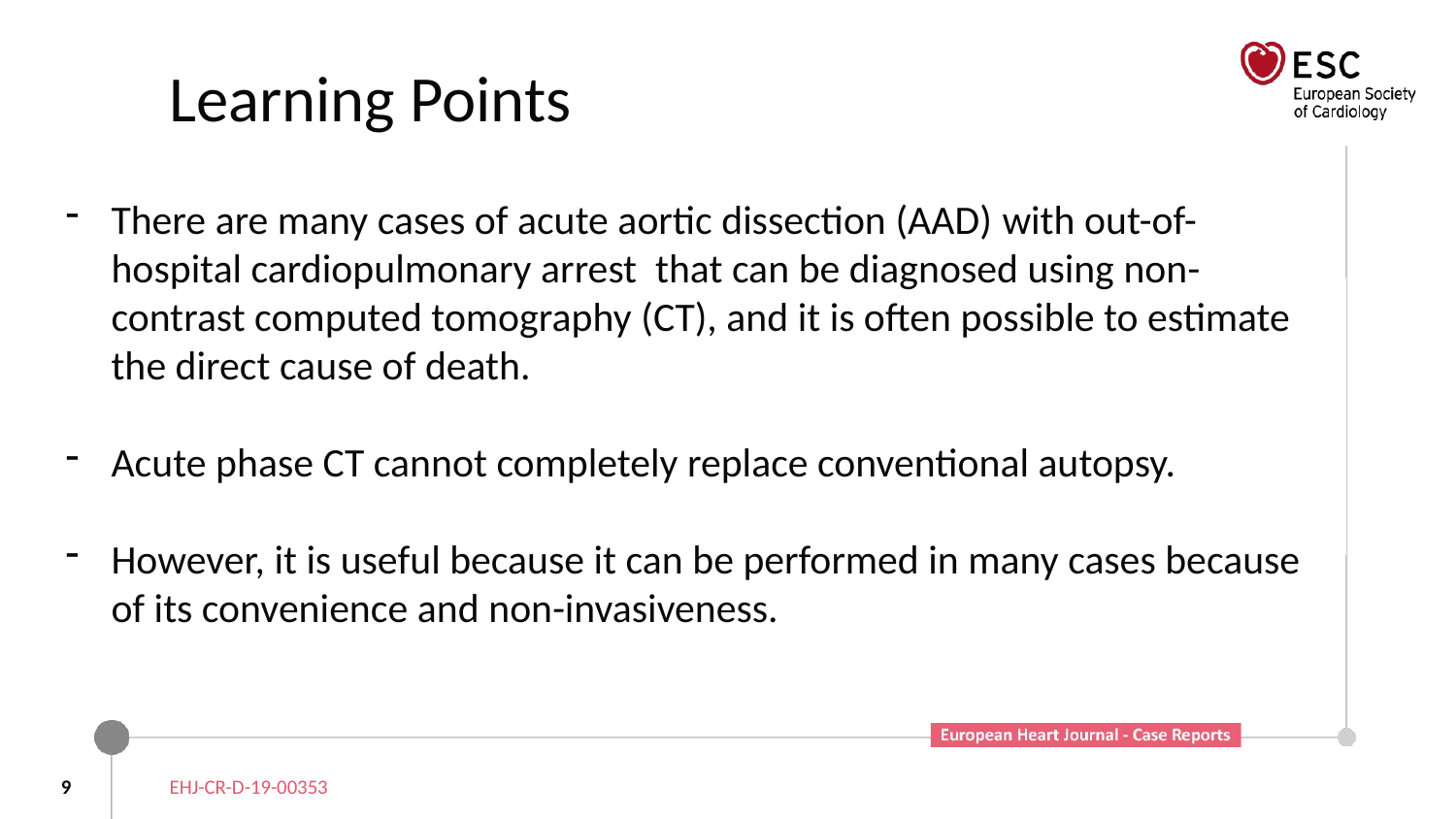

# Learning Points
There are many cases of acute aortic dissection (AAD) with out-of-hospital cardiopulmonary arrest that can be diagnosed using non-contrast computed tomography (CT), and it is often possible to estimate the direct cause of death.
Acute phase CT cannot completely replace conventional autopsy.
However, it is useful because it can be performed in many cases because of its convenience and non-invasiveness.
9
EHJ-CR-D-19-00353

## Slide 10
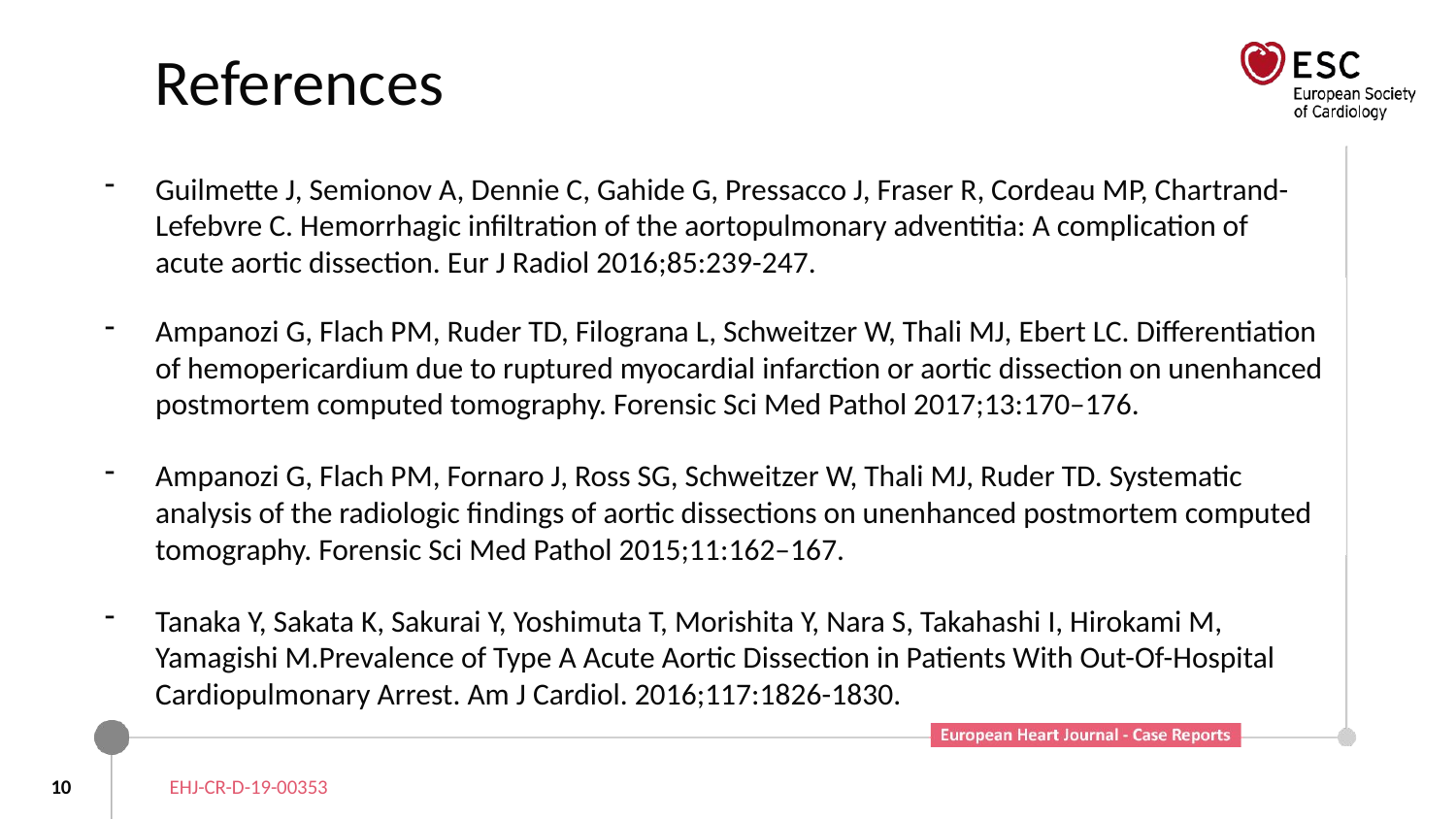

# References
Guilmette J, Semionov A, Dennie C, Gahide G, Pressacco J, Fraser R, Cordeau MP, Chartrand-Lefebvre C. Hemorrhagic infiltration of the aortopulmonary adventitia: A complication of acute aortic dissection. Eur J Radiol 2016;85:239-247.
Ampanozi G, Flach PM, Ruder TD, Filograna L, Schweitzer W, Thali MJ, Ebert LC. Differentiation of hemopericardium due to ruptured myocardial infarction or aortic dissection on unenhanced postmortem computed tomography. Forensic Sci Med Pathol 2017;13:170–176.
Ampanozi G, Flach PM, Fornaro J, Ross SG, Schweitzer W, Thali MJ, Ruder TD. Systematic analysis of the radiologic findings of aortic dissections on unenhanced postmortem computed tomography. Forensic Sci Med Pathol 2015;11:162–167.
Tanaka Y, Sakata K, Sakurai Y, Yoshimuta T, Morishita Y, Nara S, Takahashi I, Hirokami M, Yamagishi M.Prevalence of Type A Acute Aortic Dissection in Patients With Out-Of-Hospital Cardiopulmonary Arrest. Am J Cardiol. 2016;117:1826-1830.
10
EHJ-CR-D-19-00353
